# Genome Sequence of a Jumbo Bacteriophage That Infects the Kiwifruit Phytopathogen Pseudomonas syringae pv. actinidiae

**DOI:** 10.1128/MRA.00224-19

**Published:** 2019-05-30

**Authors:** Joanna K. Wojtus, Rebekah A. Frampton, Suzanne Warring, Heather Hendrickson, Peter C. Fineran

**Affiliations:** a Massey Phage Whānau, School of Natural and Mathematical Sciences, Massey University, Auckland, New Zealand; b School of Life Sciences, University of Glasgow, Glasgow, United Kingdom; c Department of Microbiology and Immunology, University of Otago, Dunedin, New Zealand; d The New Zealand Institute for Plant and Food Research Limited, Lincoln, New Zealand; e Bio-Protection Research Centre, University of Otago, Dunedin, New Zealand; Loyola University Chicago

## Abstract

Bacteriophage ϕPsa21 is a potential biocontrol agent that infects the kiwifruit phytopathogen Pseudomonas syringae pv. actinidiae. ϕPsa21 is a “jumbo” phage with a genome of ∼305 kb. Here, we present the genome sequence of ϕPsa21 and discuss potential genes indicative of the formation of nucleoid structures during viral replication.

## Abstract (precis)

Bacteriophage ϕPsa21 is a potential biocontrol agent that infects the kiwifruit phytopathogen Pseudomonas syringae pv. actinidiae. ϕPsa21 is a “jumbo” phage with a genome of ∼305 kb.

## ANNOUNCEMENT

The phytopathogen Pseudomonas syringae pv. actinidiae was identified in 1989 by Takikawa et al. as the cause of bacterial kiwifruit cankers (1). In recent years, this pathogenic bacterium has proven damaging to kiwifruit-growing regions as a pandemic strain and has spread globally (2–9). Disease management relies on an integrated control system, including agrichemicals such as copper and antibiotics, and replacement of highly susceptible varieties with more tolerant ones. There has been a growing interest in developing phage-based biocontrol strategies against plant pathogens (10, 11). Previously, we isolated multiple bacteriophages as potential biocontrol agents against P. syringae pv. actinidiae (12). In our earlier study, phage ϕPsa21 was shown to be a *Myoviridae* member, with an estimated genome size of >300 kb. Tailed phages with genomes of >200 kb are considered “jumbo” phages (11, 12).

ϕPsa21 was isolated from leaf litter in a kiwifruit orchard in Te Puke, New Zealand, and propagated on P. syringae pv. actinidiae ICMP 18800 as described earlier (12). DNA isolation and purification from ϕPsa21 were carried out using two steps as described previously (a cetyltrimethylammonium bromide method and then further purified using a Qiagen DNeasy blood and tissue kit) (12, 13). The library was prepared using an Illumina TruSeq DNA sample preparation kit (v2). MiSeq 150-bp paired-end sequencing was performed, and sequences were demultiplexed using the ea-utils suite of tools (14), resulting in ∼600× coverage. Assembly was achieved utilizing 1,241,686 reads and the default settings in the *de novo* assembler in Geneious v6.1.6; the genome was considered complete due to the coverage and since it formed a circular closed sequence, likely due to permuted ends (15). Initially, an automated annotation of the genome was produced using RAST (16), and the presence of tRNAs was determined using tRNAscan-SE (17) and ARAGORN (18). The annotation was then confirmed using DNA Master (J. G. Lawrence; http://cobamide2.bio.pitt.edu/computer.htm). A more detailed analysis of the coding sequences was achieved using BLAST (19) and PECAAN/HHPred. Annotation of the sequencing data revealed a 305,260-bp genome with a G+C content of 43.1%. Eight tRNAs and 420 protein-coding genes were annotated.

Jumbo phages have larger virions and are evolutionarily divergent compared with phages with a smaller genome (20). Recently, Chaikeeratisak et al. demonstrated that Pseudomonas chlororaphis phage 201ϕ2-1 assembles a nucleoid structure during viral infection (21). It has been hypothesized that the production of this structure is an adaptive mechanism evolved by phages to evade bacterial defense systems (21, 22). The development of the nucleoid structure by phage 201ϕ2-1 involves multiple proteins, including gp105, a major component of the nuclear shell, and PhuZ, which is a tubulin that positions the viral nucleus in the center of the infected cell (21, 23, 24). The nucleoid structure and the tubulin spindles are conserved in a number of closely related jumbo *Pseudomonas* phages (25). The ability of phages to encode these proteins is likely to indicate their potential to form nucleoids during infection. The presence of homologs of gp105 and PhuZ, combined with the large genome size and phage particle morphology, suggest inclusion in the phiKZ-like genus. Phage ϕPsa21 also shows general alignment by BLASTN with ϕKZ-like phages, namely, PhiPA3 (GenBank accession number HQ630627) aligns across 20% of the genome (maximum identity [ID], 75.7%) and Phabio (GenBank accession number MF042360) aligns across 17% of the genome (maximum ID, 73.8%). Phabio is a ϕKZ-like jumbo phage also found in New Zealand (26). Altogether, these observations place ϕPsa21in the *Myoviridae* family and *Phikzvirus* genus according to the International Committee on Taxonomy of Viruses (ICTV) classification 2018b.

Jumbo phages, including ϕPsa21, encode their own RNA polymerases, and transcription occurs independently of the host (27, 28). Lavysh et al. characterized AR9, a jumbo phage that targets Bacillus subtilis, and found that transcription relies on a multisubunit phage, RNAP (28). Analysis of the ϕPsa21 genome will contribute to future insight into the unique structural attributes and replication methods of jumbo phages and their potential as biocontrol agents.

### Data availability.

The genome sequence of *Pseudomonas* phage ϕPsa21 is available in GenBank under accession number MK552327, and the fastq files containing the 2 150-bp paired-end Illumina MiSeq reads are available in the Sequence Read Archive (SRA) under accession number PRJNA236447.
